# The relationship between prescription rates of oral corticosteroids for respiratory diseases and deprivation in England

**DOI:** 10.1038/s41533-024-00362-1

**Published:** 2024-04-25

**Authors:** Erin Barker, Jessica Pocock, Joe Moss, Nick Hex, Jordan Rankin, Richard Hudson

**Affiliations:** 1 York Health Economics Consortium, Enterprise House, Innovation Way, University of York, Heslington, York, YO10 5NQ UK; 2 Sanofi, 410 Thames Valley Park Dr, Earley, Reading, RG6 1PT UK

**Keywords:** Asthma, Chronic obstructive pulmonary disease

## Abstract

Respiratory diseases, including asthma and chronic obstructive pulmonary disease (COPD), are common in England with the worst respiratory outcomes observed in the most deprived areas. There is limited published research to establish whether the rate of oral corticosteroid (OCS) prescribing for asthma and COPD is linked to levels of deprivation. This study carried out a multivariable regression analysis of publicly available data and found that deprivation is associated with a statistically significant increase in the proportion of patients receiving an OCS prescription for asthma or COPD at a GP practice level (p < 0.001). The model estimated that the proportion of prescriptions is 1.88% (95% CI 1.83% to 1.92%) and 2.84% (95% CI 2.70% to 2.98%) for the least deprived GP practice and the most deprived GP practice, respectively. This study lays the groundwork for future research using individual patient level data to consider the impact of variation in OCS prescribing rates.

## Introduction

Respiratory diseases are common in England, with approximately one in five people affected, which places a significant burden on individuals and the health and social care system^1^. Two of the most common respiratory diseases are asthma and chronic obstructive pulmonary disease (COPD)^2^. The Global Initiative for Asthma defines asthma as a highly variable chronic condition, characterised by inflammation and obstruction of the airways. The intensity of airway obstruction can vary over time and result in episodic symptoms, including wheezing, a tight chest, coughing and breathlessness^3^. The Global Initiative for Chronic and Obstructive Lung Disease defines COPD as an often progressive, heterogenous lung condition that involves abnormal airways and / or alveoli. Airway and alveoli abnormalities may be because of bronchitis / bronchiolitis and emphysema, respectively, for example. This results in obstructions to airflow and causes symptoms such as coughing, dyspnoea, expectoration, and exacerbations^4^.

Asthma and COPD are both characterised by exacerbations, which may lead to hospitalisation and death^1^. Although both diseases are not curable, various treatments may help to improve control, prevent exacerbations and recurring symptoms (e.g. shortness of breath), as well as slow down the progression of COPD^1,2,5^. People with asthma and COPD in England are usually offered a variety of inhalers to relieve symptoms and prevent exacerbations. However, depending on the severity of symptoms, oral corticosteroids (OCS) may be prescribed^6–8^. People with asthma may need to be prescribed an OCS, most commonly prednisolone, to manage exacerbations or severe episodes of symptoms, but they are not recommended for long-term use^9^. The National Institute for Health Care and Excellence (NICE) guideline NG115 outlines that people with COPD may also need to be prescribed an OCS to manage severe episodes of symptoms, but they are, again, not recommended for long-term use for COPD^7^.

Repeated and long-term use of OCS is associated with serious health risks for asthma and COPD, co-morbidities such as osteoporosis and cardiovascular disease, as well as increased mortality risk for people with COPD^8–11^. Despite these health concerns, people with asthma and COPD often remain on OCS instead of being moved on to an alternative treatment. For asthma, biologic treatments have been shown to have better patient outcomes and fewer side effects^11^. Whilst there are currently no licenced biologics for the treatment of COPD several are under investigation^12–14^. In the treatment of asthma, long-term use of OCS has been estimated to range from 40 to 60% in the literature with great variation in rates reported between countries^15^. A previous study based on a Danish cohort of people with COPD who were treated with an OCS found that 41% of the cohort were on long-term OCS treatment^16^. In the UK, OCS prescribing remains high for asthma with approximately 1.29 million people receiving a prescription for OCS every year and 140,000 people being prescribed two or more courses^10,17^.

Previous studies have shown there to be an association between incidence rates of respiratory diseases and areas of higher deprivation, where people have the greatest exposure to risk factors for exacerbation of symptoms (e.g. treatment adherence and smoking) and, therefore, have higher incidence of asthma and COPD^18–20^. According to a 2019 survey carried out by Asthma UK, people on lower incomes have more severe asthma symptoms, more visits to emergency care and greater use of OCS than people on higher incomes^18^. A retrospective analysis carried out by Chalitsios et al. found that asthma and COPD were significantly associated with OCS prescriptions^10^. Bisphosphonate (BP) is prescribed as a first-line treatment for glucocorticoid-induced osteoporosis, an adverse effect associated with OCS treatment. However, only COPD was associated with BP prescriptions. The study also suggested that prescribing patterns of OCS and BP for asthma and COPD in the UK were linked to deprivation^10^. The study found that the most deprived areas were less likely to prescribe both OCS and BP than the least deprived areas which may reflect a marked difference in access to care. The difference in findings between qualitative and quantitative evidence in the direction of the relationship between OCS use and deprivation may be linked to the limitations in the quantitative data available. In addition, some confounders could not be accounted for in the Chalitsios et al. study and the inclusion of BP may have contributed to these findings.

Therefore, we carried out a study to assess the relationship between prescription rates of prednisolone for people with asthma or COPD and deprivation in England. The study used publicly available aggregate data at the GP practice level in England and, therefore, did not require ethical approval.

## Results

### GP practice characteristics

A total of 6830 GP practices were identified. Of those, 442 GP practices had missing data. Preliminary analysis indicated significant outliers based on Cook’s distance and standardised residuals. In total, 323 GP practices were identified as outliers. Hence, 765 GP practices were excluded from the analysis (11.2%) resulting in a total of 6065 GP practices in the base case analysis. Excluding observations defined as outliers improved the model fit based on the pseudo-R-squared value (0.5442 to 0.6969). The preliminary model results (i.e. including all data points considered outliers) and diagnostic plots can be found in Supplementary Table 2 and Supplementary Figure 3 (and Supplementary Figure 4 excludes all data points considered outliers). The GP practice characteristics used in the final analyses are summarised in Table 1.

**Table 1:** GP practice characteristics.

Characteristics of GP^a^ practices	Mean (SD^b^) N = 6065
IMD^c^ score	22.78 (11.26)
Age	40.37 (4.52)
Proportion of males (%)	50.11 (1.97)
Proportion of prescriptions (%)	2.26 (0.95)
Proportion of patient’s adherence to treatment (%)	54.71 (7.55)
Prevalence of arthritis (%)	0.78 (0.26)
Prevalence of asthma (%)	6.49 (1.38)
Prevalence of COPD^d^ (%)	1.97 (0.90)
Prevalence of mental health issues (%)	0.94 (0.37)
Prevalence of obesity (%)	10.78 (3.83)
Prevalence of smoking (%)	14.32 (5.93)

^a^GP – general practitioners;

^b^SD – standard deviation;

^c^IMD – index of multiple deprivation;

^d^COPD – chronic obstructive pulmonary disease.

### Base case: prednisolone prescribing and deprivation

The regression model indicates that for a one-unit increase in IMD score, there is a statistically significant absolute increase of approximately 0.016% (p < 0.001) in the proportion of all patients at a GP practice prescribed prednisolone, when all other variables are held constant. Furthermore, as age increases by one year or the prevalence of asthma, arthritis, COPD, and smoking increased by 1%, there is a significant absolute increase in the proportion of patients prescribed prednisolone while also receiving treatments for asthma or COPD of approximately 0.025% (p < 0.001), 0.990% (p < 0.001), 0.812% (p < 0.001), 0.985% (p < 0.001) and 0.005% (p < 0.001), respectively, when all other variables are held constant. Conversely, as the proportion of males, the prevalence of mental health conditions or treatment adherence increases by 1%, there is a statistically significant absolute decrease in the proportion of patients prescribed prednisolone while also receiving treatments for asthma or COPD of approximately 0.059% (p < 0.001), 0.023% (p < 0.001), and 0.009% (p < 0.001), respectively, when all other variables are held constant. Prevalence of obesity was associated with an increase in prescribing of prednisolone, but this was not statistically significant (p = 0.289). Note, as the statistical model is on the log scale, all probabilities presented are approximate due to the non-linear nature of the model. The model coefficients are shown in Table 2.

**Table 2:** Model coefficients for the base case analysis.

Variable	Log Odds	SE^a^	P-Value
Intercept	−4.21	0.133	<0.001
IMD^b^	0.01	0.001	<0.001
Age	0.01	0.001	<0.001
Males	−2.32	0.212	<0.001
Treatment adherence	−0.40	0.060	<0.001
Prevalence of arthritis	5.47	1.666	0.001
Prevalence of asthma	12.89	0.310	<0.001
Prevalence of COPD^c^	17.10	0.598	<0.001
Prevalence of mental health	−7.54	1.098	<0.001
Prevalence of obesity	0.11	0.108	0.289
Prevalence of smoking	0.24	0.069	<0.001

R-squared of the model: 0.6969.

^a^SE – standard error.

^b^IMD – index of multiple deprivation.

^c^COPD – chronic obstructive pulmonary disease.

**Fig. 1:**
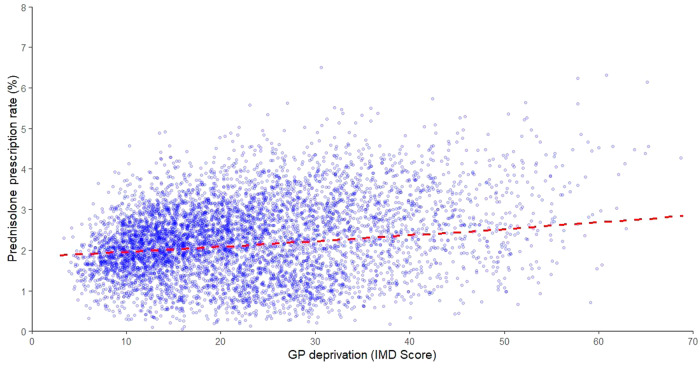
Prednisolone prescription rate for asthma or COPD. The scatter plot shows the trend of prednisolone prescription rate for the entire GP practice population by index of multiple deprivation (IMD) score. The red line represents the model prediction for the average GP practice.

Model predictions indicate that the proportion of prescriptions for the GP practice with the lowest deprivation score is 1.88% (95% CI 1.83% to 1.92%), with the highest deprivation score is 2.84% (95% CI 2.70% to 2.98%), and at the mean deprivation score is 2.12% (95% CI 2.10% to 2.15%), when assuming all other variables remain constant. This represents a 1.51-fold increase in the proportion of all patients at a GP practice prescribed prednisolone while also receiving treatments for asthma or COPD for the GP practice with the highest deprivation, compared with the GP practice with the lowest deprivation. Figure 1 shows the trend of prednisolone prescribing by IMD score.

### Sensitivity analysis: age-restricted variables

Using the same data as the base case but excluding adult-based confounders, the beta regression shows for each unit increase in IMD there is a 0.01 increase in the log-odds of being prescribed prednisolone, when all other variables are held constant. GP practices with a higher prevalence of asthma or COPD, and GP practices with a higher proportion of older patients were associated with a statistically significant increase in prescribing of prednisolone. GP practices with a higher proportion of males, or higher prevalence of mental health, and higher treatment adherence were associated with a statistically significant decrease in prescribing of prednisolone. The model coefficients are shown in Table 3. The diagnostic plots are presented in Supplementary Figure 3.

**Table 3:** Model coefficients for all age’s data excluding ‘adult’ based confounders.

Variable	Log Odds	SE^a^	P-Value
Intercept	−4.16	0.131	<0.001
IMD^b^	0.01	0.001	<0.001
Age	0.01	0.001	<0.001
Males	−2.31	0.211	<0.001
Treatment adherence	−0.42	0.060	<0.001
Prevalence of asthma	13.04	0.301	<0.001
Prevalence of COPD^c^	17.82	0.570	<0.001
Prevalence of mental health	−7.47	1.084	<0.001

R-squared of the model: 0.6951;

^a^SE – standard error;

^b^IMD – index of multiple deprivation;

^c^COPD – chronic obstructive pulmonary disease.

The preliminary model results for all confounding variables using the ‘adult’ only dataset indicated significant outliers based on Cook’s distance and standardised residuals. A total of 331 GP practices were identified as outliers and removed from the analysis. The diagnostic plots are presented in Supplementary Fig. 4 (including outliers) and Supplementary Fig. 5 (excluding outliers).

After removing outliers, the beta regression suggests that a one unit increase in IMD score results in a 0.01 change in the log-odds of being prescribed prednisolone, when all other variables are held constant. GP practices with a higher proportion of older patients, or a higher prevalence of asthma or COPD or smoking were associated with a statistically significant increase in prescribing of prednisolone. Whereas GP practices with a higher proportion of males, or higher treatment adherence, or a higher prevalence of mental health conditions, or a higher prevalence of obesity were associated with a statistically significant decrease in prescribing of prednisolone. Prevalence of arthritis was associated with an increase in prescribing of prednisolone but was not statistically significant. The coefficients are presented in Table 4.

**Table 4:** Model coefficients for ‘adults’ only data including all confounders.

Variable	Log Odds	SE^a^	P-Value
Intercept	−4.58	0.124	<0.001
IMD^b^	0.01	0.0005	<0.001
Age	0.02	0.001	<0.001
Males	−2.13	0.170	<0.001
Treatment adherence	−0.35	0.057	<0.001
Prevalence of arthritis	0.87	1.596	0.5846
Prevalence of asthma	11.73	0.296	<0.001
Prevalence of COPD^c^	16.12	0.558	<0.001
Prevalence of mental health	−7.98	1.037	<0.001
Prevalence of obesity	0.24	0.102	0.020
Prevalence of smoking	0.32	0.065	<0.001

R-squared of the model: 0.7231;

^a^SE – standard error;

^b^IMD – index of multiple deprivation;

^c^COPD – chronic obstructive pulmonary disease.

The sensitivity analyses indicate that adjusting for age (i.e. restricting the data to adults only) results in a small change in the findings of the study results.

## Discussion

This study has shown that an increase in IMD score (i.e. an increase in deprivation) is associated with a statistically significant increase in the proportion of patients prescribed prednisolone while receiving treatment for asthma or COPD at GP practice level in England while controlling for confounding variables.

The results of the current study are similar to those reported in the 2019 survey by Asthma UK which identified that people on lower incomes reported greater use of OCS than people on higher incomes^18^. These findings highlight health inequalities for people with asthma and COPD which may lead to an increase in OCS prescriptions in more deprived areas. Previous qualitative work by Asthma UK found that many people are not receiving basic asthma and COPD care, and that this is further exacerbated by health inequalities^18,19,21,22^. Similarly, a previous study reported that more deprived areas are associated with a greater environmental burden^23^. This could influence the incidence or severity of respiratory disease in these areas, which may lead to an increase in OCS prescription^23^.

In contrast, however, a similar retrospective analysis by Chalitsios et al. found that people attending GP practices in the most deprived areas were less likely to be prescribed both OCS and BP than the least deprived areas^10^. The paper suggests that the differences in OCS prescribing between IMD quintiles may indicate health inequalities in terms of access to treatment^10^. The difference in findings in this study may be due to differences in the study methodology. This analysis was focused on the OCS prednisolone whereas the study conducted by Chalitsios et al. included several different OCS sourced from OpenPrescribing database^10^ and used that data to estimate the number of items prescribed per 1000 rather than the proportion of unique patients receiving a prescription. Furthermore, the prescribing data were from 2018 and IMD 2015 was used in the Chalitsios study.

A strength of the current study was the inclusion of the effects of confounding variables in the analysis, identified by clinical experts. Inclusion of confounding variables allows for greater certainty of the true relationship between OCS prescribing for asthma and COPD and social deprivation. The analysis demonstrated that confounding variables were related to the prescribing of prednisolone. GP practices with a higher prevalence of asthma, arthritis, COPD, or smoking were associated with a statistically significant increase in prescription rates of prednisolone. Whereas GP practices that contained more males, or a higher prevalence of mental health, or higher treatment adherence were associated with a decrease in prescribing prednisolone. Prevalence of obesity was associated with a statistically significant increase in prescribing of prednisolone, but only after controlling for age-restricted variables. Another strength of the analysis is the use of real-world datasets for the period of interest (2019 to 2020).

Some important limitations should be considered when interpreting the results of the analysis. The analysis was carried out using aggregate population data (i.e. GP practice level) that may not show the full effect of the relationship. The impact of confounders may be distorted because aggregate data can flatten out effects that might be observed in a patient-level dataset. It was not feasible to use patient-level data for the current analyses and, therefore, the limitations of using publicly available aggregated data are acknowledged. Nevertheless, the results suggest that there is a statistically significant association between the most deprived GP practices prescribing higher levels of OCS compared with the least deprived GP practices.

Another potential limitation of the analysis was age because it could not be matched across the datasets and/or variables. However, the results of sensitivity analysis (i.e. excluding ‘adult’ only variables or restricting the data based on age) demonstrate that age has a limited impact on the overall results.

It was not possible to differentiate between prescriptions for asthma and prescriptions for COPD, due to the granularity of the data. However, the results suggest that an increase in either prevalence of asthma or COPD are associated with an increase in prescribing of prednisolone. Prednisolone prescriptions may have been overestimated because it was not possible to identify within the data the medical condition prednisolone was prescribed to treat in people receiving a medication for asthma and COPD^24^. Therefore, some of these prescriptions may have been prescribed to treat other acute medical conditions^24^.

Data were not available at a GP practice level for some of the potential confounding variables which may affect the results. There were 442 practices with missing data which may potentially bias the results. Finally, GP practices identified as outliers were excluded from the analysis. Generally, GP practices identified as outliers had unusually high or low values for a variable(s) in the datasets, for example, one GP practice had a substantially higher mean age of 80 compared to the average age of 40.37 for all GP practices. This may be due to large differences in patient characteristics across practices or may have occurred due to data entry error. Overall, exclusion of outliers improved the model fit (demonstrated by an increase in the R-squared value of 0.5442 to 0.6969).

The UK Government has a growing health policy focus on reducing healthcare inequalities, with local health commissioners being directed to address these issues^25^. Alongside this, the Government has established a Major Conditions Strategy to address the burden of disease of long-term conditions, including chronic respiratory diseases^26^. This study highlights that OCS prescription rates vary by GP practices, with higher levels of deprivation associated with higher GP practice OCS prescription rates, in contrast to previous research. This finding warrants further investigation, due to the adverse health effects associated with using OCS for asthma and COPD, into whether OCS prescriptions could be minimised in areas with higher prescription rates^8–10^. As well as whether these differences can be attributed to health inequalities. Identifying the reasons for the higher prednisolone prescription rates by GP practice deprivation will be key in minimising potential health inequalities and whether these rates can be reduced.

Future research should be carried out to examine the relationship between OCS use for asthma or COPD and social deprivation using individual patient data to explore the differences in OCS prescription rates further. This would provide detailed prescription data for individual patients, including other OCS, and allow the separate analysis of different respiratory disease areas. Furthermore, IMD scores at a granular level, rather than aggregated to GP practices, would result in more robust analyses. Finally, variables that might impact the relationship, but were not available at a GP practice level, could potentially be obtained from individual patient data.

This study found a positive relationship between OCS (prednisolone) prescribing for asthma or COPD and social deprivation at GP practice level in England. However, further research should be carried out at an individual patient level for more granular findings.

## Methods

### Study design

This study was a retrospective, observational study using publicly available secondary data. To account for the potential impact of the Covid-19 pandemic on OCS prescription rates, data were extracted for the year April 2019 to March 2020, alongside the 2019 index of multiple deprivation (IMD) scores. The study population was GP practices in England. GP practices were excluded from the analysis if data were not available. Regression analysis was used to explore the relationship between patients receiving an OCS prescription for asthma or COPD and deprivation at a GP practice level.

### Data sources

Data were obtained from the NHS Business Services Authority (NHSBSA) respiratory dashboard, Quality and Outcomes Framework (QOF), Fingertips Public Health Database (PHD), and NHS Digital^24,27–29^.

The NHSBSA respiratory dashboard aims to allow healthcare providers to monitor and improve prescribing for respiratory care^24^. The dashboard focuses on asthma and COPD and includes seven comparator measures. While individual patient data are used to derive the measures, personally identifiable data are not included. Hence, measures are available at the GP practice level and aggregated to the Clinical commissioning Group (CCG) level. In addition, the data does not identify whether the OCS prescription is for asthma or COPD and therefore the data cannot be separated to consider the OCS prescriptions for individual diseases. Prescribing data includes all items prescribed in primary care but does not include hospital prescribing.

Fingertips PHD is a large collection of public health datasets sourced from government and NHS agencies (e.g. the Office for National Statistics and NHS Digital)^27^. Fingertips PHD was developed to support healthcare professionals and local authorities in providing effective and appropriate healthcare and to reduce health inequalities in their area.

NHS Digital collects patient data to support researchers and the NHS to understand diseases and improve healthcare services^28^. Each month, NHS Digital provides patient demographic data at the GP practice level including the number of patients registered at a GP practice stratified by age and gender.

The QOF is a programme that aims to support a high quality of care by rewarding good practices and setting targets for GP practices to achieve^29^. Practice achievements are measured by practices providing the QOF with data on several outcome indicators, including public health factors. The QOF only collects data from GP practices that participate in the voluntary scheme.

All data from all sources were obtained at the GP practice level and linked by a unique area code.

### Outcome and exposure variables

The primary outcome variable was the proportion of patients receiving a prednisolone prescription. This was calculated as the number of unique patients receiving a prednisolone prescription (of any dosage) while also receiving medication to treat asthma or COPD divided by the total number of patients for each practice. Prescription data were obtained from the NHSBSA respiratory dashboard.

The exposure variable was social deprivation. IMD score was used as a proxy for social deprivation. IMD scores are calculated for small areas in England called Lower-layer Super Output Areas (LSOAs) and are based on seven domains: income; employment; education; skills and training; health and disability; crime; barriers to housing services and living environment^30^. Scores at the GP practice level are estimated using a population weighting method, with higher scores representing more deprived areas. The 2019 IMD scores at the GP practice level were obtained from Fingertips PHD.

### Confounding variables

A confounding variable is a variable that influences both the outcome and exposure variable. For example, people who live in areas with higher levels of deprivation may have a higher prevalence of obesity, which may increase patient risk of developing asthma and therefore result in increased management of respiratory diseases using OCS. Confounding variables that may generate bias in the analysis were identified by interviewing three respiratory clinicians via web-based conferencing. All confounder variables were reviewed to identify which could feasibly be included in the analysis. Confounding variables included in the analysis were age, sex, treatment adherence, and prevalence of asthma, COPD, rheumatoid arthritis, mental health conditions, obesity, and smoking. Examples of potential confounding variables identified by clinicians that could not be included in the analysis were accident and emergency (A&E) attendance rates, housing density, access to health care, patient understanding of asthma, quality of the indoor living environment, ethnicity, prevalence of depression, prevalence of osteoporosis, and areas with high pollution. The exclusion criteria for variables were a) data were not publicly available b) variables could not feasibly be matched (for example, age-restricted data of 50 + ), and c) data could not be linked to GP practice data. A summary table of excluded variables are presented in Supplementary Table 1.

Mean age was estimated based on grouped data. The number of patients in each age category was multiplied by the midpoint of the age category. These values were summed and divided by the total number of patients to provide an estimated mean age for each GP practice. The proportion of males was derived as the total number of males divided by the total number of patients for each GP practice. Demographic variables were sourced from NHS Digital based on data from March 2020.

The proportion of patients prescribed five or fewer steroid inhalers, including inhaled corticosteroids (ICS) and long-acting beta-agonists (LABA) products, was used as a proxy for treatment adherence. This metric was obtained directly from the NHSBSA respiratory dashboard. The NHSBSA specifies that people who collect five or fewer prescriptions may benefit from a medication review and, therefore, may not be adhering to treatment^24^. The proportion of patients was calculated as the number of patients who have collected five or fewer prescriptions divided by the total number of patients receiving any prescriptions for asthma or COPD.

Prevalence data were obtained for asthma, COPD, rheumatoid arthritis, mental health, obesity, and smoking by GP practice. These variables were obtained from the QOF database, apart from smoking prevalence which was extracted from Fingertips PHD. Smoking prevalence is sourced from the GP patient survey (GPPS), an annual survey sent to patients in the UK to collect data on patient opinions of their GP practice as well as patient information such as smoking status. Smoking prevalence is the proportion of people who responded ‘occasional smoker’ or ‘regular smoker’ in the 2020 GPPS. Prevalence of mental health conditions is the proportion of patients registered at a GP practice with schizophrenia, bipolar affective disorder, or other psychoses as well as any patients receiving lithium treatments^29^. At source, some prevalence variables were age restricted: obesity prevalence was based on patients aged 18 and over; rheumatoid arthritis prevalence was based on people aged 16 and over; asthma prevalence was based on people aged six and over; and smoking prevalence was based on people aged 15 and over.

### Statistical analysis

Data were analysed using descriptive statistics and continuous variables were summarised as mean and standard deviation. Multivariate beta regression was used to evaluate the association between prescription rates of prednisolone for patients with asthma or COPD and social deprivation after adjusting for confounders including age, sex, and treatment adherence in addition to the prevalence of rheumatoid arthritis, asthma, COPD, mental health conditions, obesity, and smoking. The assumptions of the beta regression were assessed using diagnostic plots such as Cook’s distance plot (see supplementary material for further details).

As part of the statistical model validation checks, outliers were identified based on Cook’s distance and the size of the standardised residuals. An observation was considered an outlier if it had a Cook’s distance greater than 4/n, where n is the number of observations^31^. Additionally, an observation with a standardised residual that was larger than 3 (in absolute value) was also considered an outlier^31^.

Due to the non-linear nature of the statistical model, the regression coefficients cannot be directly translated into absolute percentage changes in prescription levels. Therefore, to generate an approximate impact on prescriptions, the regression outputs were used to predict the average level of prescription whilst keeping all variables held constant at their mean value. Each variable was then set to 0 and 100 (or 0 and 1 depending on their scale) and the difference between the highest and lowest value divided by 100. This produces a simplified linear prediction on how a 1-unit change in the variable impacts the absolute proportion of patients in a GP practice who receive the prescription.

Furthermore, in order to generate 95% confidence intervals (CIs) for the regression predictions a bootstrapping approach was taken. This involves re-running the statistical model on a subset of the data 10,000 times to calculate the level of uncertainty present within the model predictions.

Log odds, standard error, and P-values were presented from the multivariable beta regression models. P-values < 0.05 were considered statistically significant. All analyses were conducted using R version 4.2.1^32^.

### Sensitivity analysis

Variables that applied an age restriction were considered ‘adult’ variables (i.e. age restricted data of 15 + ). ‘Adult’ variables included prevalence of obesity, rheumatoid arthritis, and smoking. Sensitivity analysis was conducted to explore the impact of age-restricted variables on the results of the analysis by running two additional models. Firstly, using the same data used in the base case model, a model was run that excluded ‘adult’ variables. Following this, an ‘adult’ only dataset was created by excluding patients based on age. Since age categories were defined differently across data sources, the same age limits could not be applied to all variables. For prescription data, age categories “0 to 5”, “6 to 11”, and “12 to 15” were excluded from the analysis. For patient demographic data, age categories “0 to 4”, “5 to 9”, and “10 to 14” were excluded from the analysis. A final model was run that included all confounding variables using the ‘adult’ only dataset. Therefore, variables in the third model only included people aged 15 plus to create an ‘adult’ only population. For the ‘adult’ only dataset, observations defined as outliers were also excluded from the analysis.

### Reporting summary

Further information on research design is available in the Nature Research Reporting Summary linked to this article.

### Supplementary information

Supplementry Material

Reporting Summary

## Data Availability

The datasets used in the current study are publicly available from the QOF (https://digital.nhs.uk/data-and-information/publications/statistical/quality-and-outcomes-framework-achievement-prevalence-and-exceptions-data/2019-20), NHSBSA respiratory dashboard (https://www.nhsbsa.nhs.uk/access-our-data-products/epact2/dashboards-and-specifications/respiratory-dashboard), Fingertips PHD (https://fingertips.phe.org.uk), and NHS Digital (https://digital.nhs.uk/data-and-information/publications/statistical/patients-registered-at-a-gp-practice/march-2020). The approach taken in the analysis is outlined in the main text and could be reproduced in any similar retrospective study.

## Code availability

Code can be accessed upon reasonable request from the corresponding author.
