# Supplementary material for: The relationship between prescription rates of oral corticosteroids for respiratory diseases and deprivation in England
Source: NPJ Prim Care Respir Med. 2024 Apr 25;34:3. doi: 10.1038/s41533-024-00362-1 (PMC11045771; doi:10.1038/s41533-024-00362-1)
Supplement: Supplementary file 1 — Supplementry Material [file 41533_2024_362_MOESM1_ESM.pdf]

Supplementary Table 1

Variables	Reason for exclusion
Areas of high pollution	It is not possible to link air pollution data to GP practices. This variable would be more suitable for local authority or CCG level data.
Areas with high A&E attendance	It is not possible to link A&E admissions to GP practices using publicly available data. This variable would be more suitable for patient level data.
Patients further away from hospital	A variable indicating the distance to nearest hospital from the GP practice was not readily available. Furthermore, distance from hospital for individual patients would require patient level data. It may be possible to compute a distance from each GP practice to a major asthma centre, but this would be very time consuming and beyond the scope of the study.
Housing density	No suitable variables were found that indicated housing density. This is expected to be captured, to an extent, in the IMD measure through the 'living environment' domain.
Access to health care	The clinicians all referred to access to health care being an issue in one form or another (i.e. access to A&E, doctors, prescriptions, asthma care, etc.). No suitable variables were found to accurately measure or represent access to health care at a GP practice level.
Education (understanding asthma)	No suitable variables were found that indicated knowledge of asthma.
Quality of indoor living environment	No suitable variables were found that indicated quality of indoor living environment. This is expected to be captured, to an extent, in the IMD measure through the 'living environment' domain.
Ethnicity (language barrier)	It is not possible to link ethnicity data to GP practices using publicly available data. This variable would be more suitable for analysis at CCG level.
Depression	It is not possible to link depression data to GP practices using publicly available data. This variable would be more suitable for CCG level analysis.
Osteoporosis i.e. fractures	The QOF contains prevalence of osteoporosis but only for people aged 50 and over.

Supplementary Table 2

Variable	Log Odds	SE	P-Value
Intercept	-3.71	1.524	<0.001
IMD	1.01	6.156	<0.001
Age	1.78	1.459	<0.001
Males	-3.26	2.352	<0.001
Treatment adherence	-4.63	7.141	<0.001
Prevalence of arthritis	1.88	1.977	0.924
Prevalence of asthma	1.05	3.332	<0.001
Prevalence of COPD	1.59	7.061	<0.001
Prevalence of mental health	-1.21	9.098	<0.001
Prevalence of obesity	-7.17	1.230	<0.001
Prevalence of smoking	1.09	8.253	0.186

R-squared of the model: 0.5442; COPD – chronic obstructive pulmonary disease; IMD - index of multiple deprivation; SE – standard error.

Supplementary Methods

Diagnostic plots are presented in the supplementary figures below. The diagnostic plots are presented to demonstrate whether the model assumptions were met and the subsequent changes in the data after removing outliers and residuals. The following diagnostic plots are presented a) the Residuals vs indices plot is used to check the homoscedasticity assumption has been met by producing a random scatter and equal spread of data points around the residual line; b) Cook's distance plot shows the influence of observations on the model predictions, and outliers would be expected if the lines are not of a relatively similar length and distribution; c) the Generalized leverage vs predicted values plot is a second check for influential observations. Large leverage values potentially indicate the presence of outliers; and d) the Residuals vs linear predictor plot indicates whether the linearity and homoscedasticity assumptions have been met if there is a random scatter and equal spread of data points around the residual line.

Supplementary Figure 1

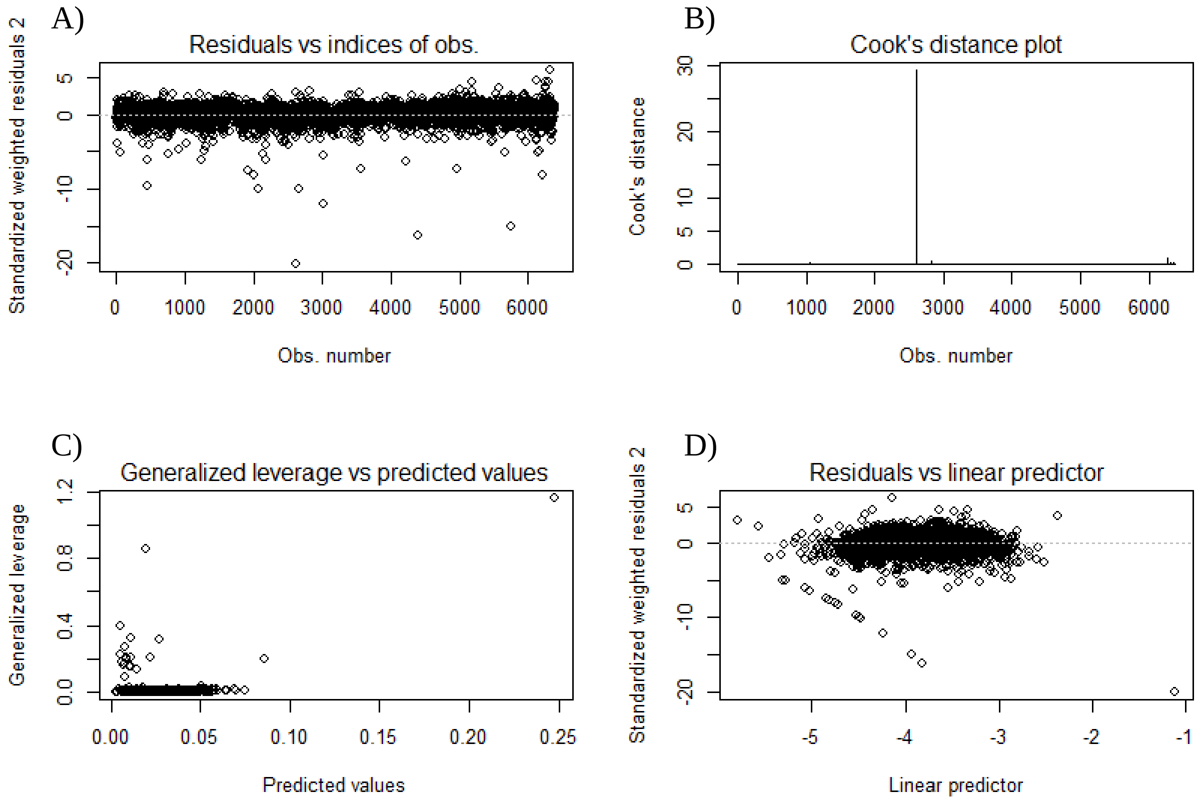

See supplementary methods for further details on the diagnostic plots.

Supplementary Figure 2

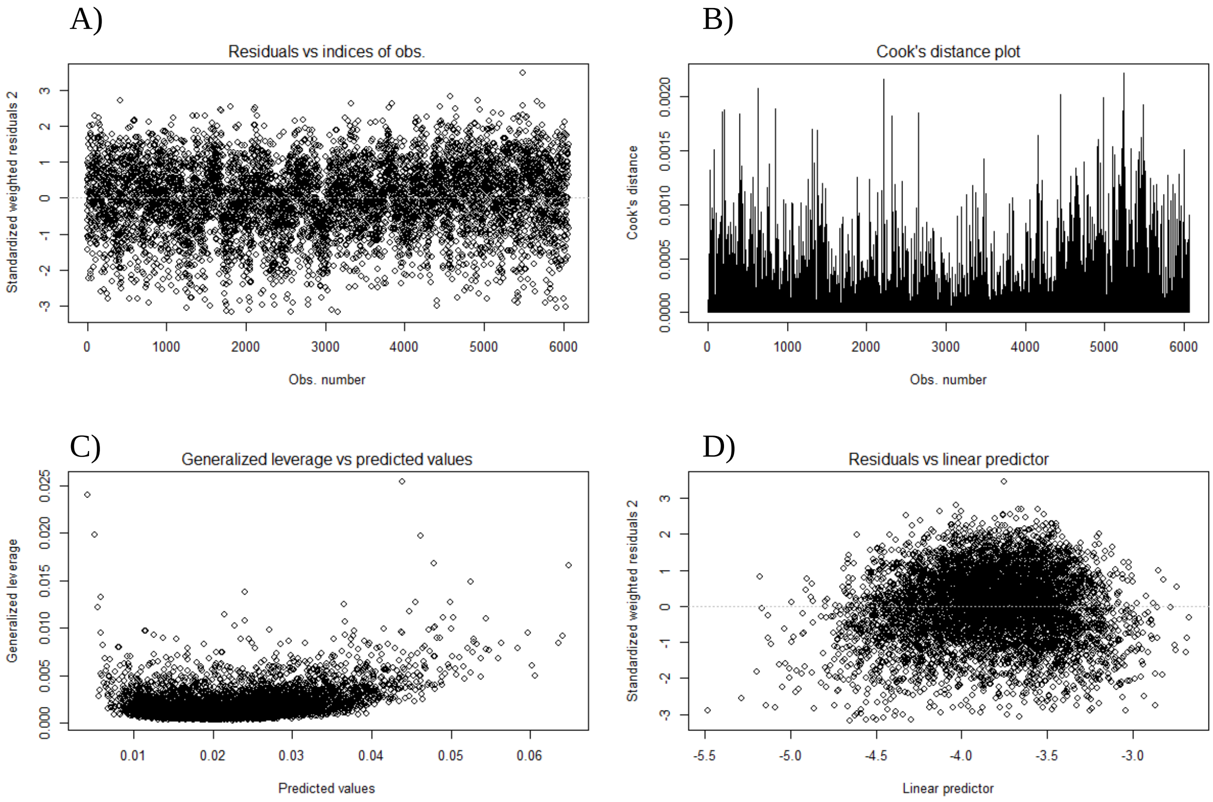

See supplementary methods for further details on the diagnostic plots.

Supplementary Figure 3

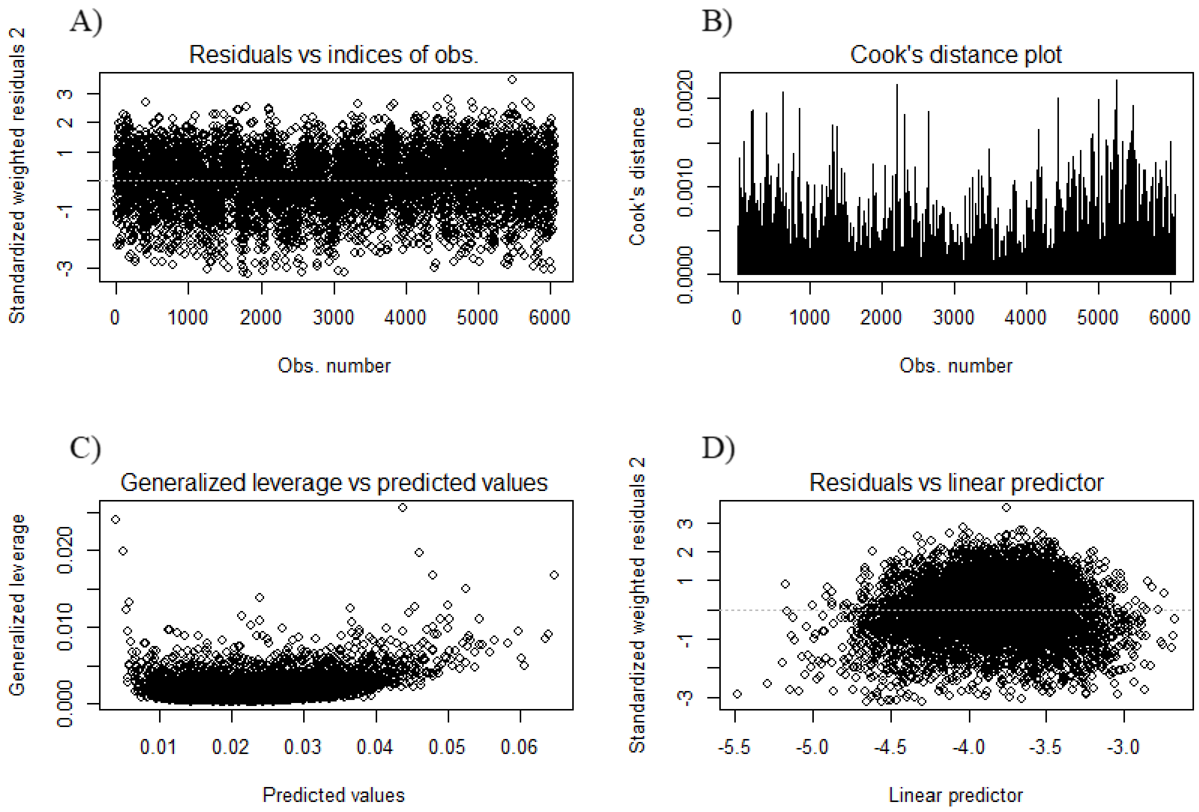

See supplementary methods for further details on the diagnostic plots.

Supplementary Figure 4

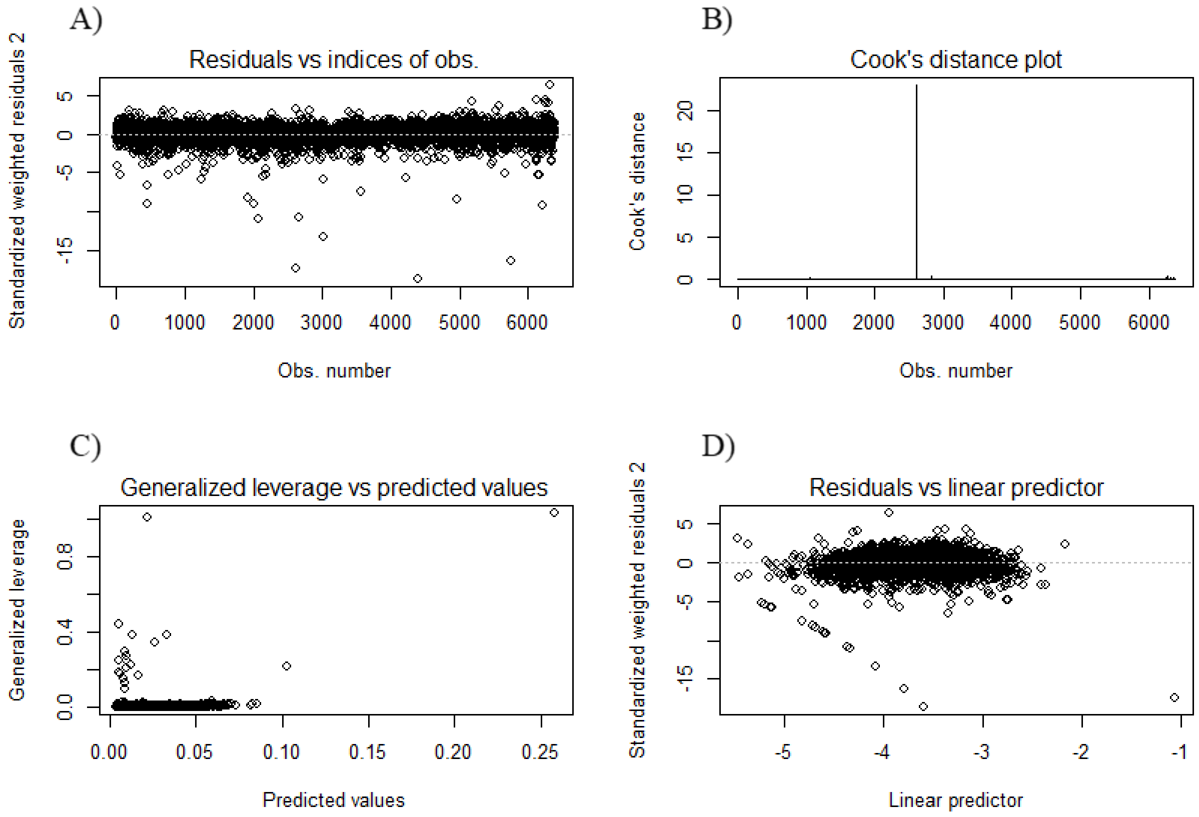

See supplementary methods for further details on the diagnostic plots.

Supplementary Figure 5

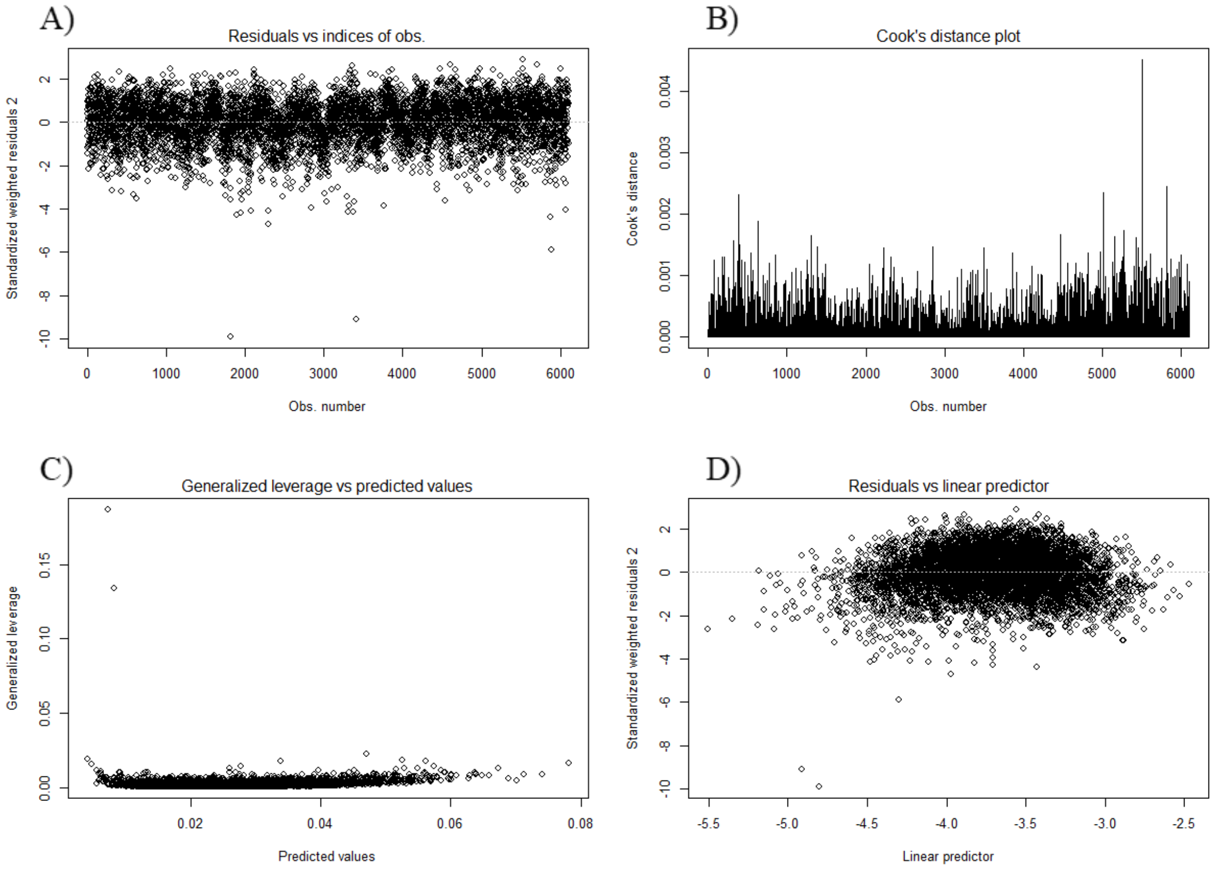

See supplementary methods for further details on the diagnostic plots.
